# Supplementary material for: Acceptability and feasibility of using contingency management for cannabis reduction in specialist mental health services for psychosis: A qualitative study of staff views
Source: PLoS One. 2023 Feb 8;18(2):e0281368. doi: 10.1371/journal.pone.0281368 (PMC9907825; doi:10.1371/journal.pone.0281368)
Supplement: Attachment — Submitted filename: response to PLOS One 22-11-2022.docx [file pone.0281368.s001.docx]

PONE-D-21-36088R1
Acceptability and feasibility of using contingency management for cannabis reduction in specialist mental health services for psychosis: A qualitative study of staff views
Dr Luke Sheridan Rains

Dear Dr. Sheridan Rains,

We've checked your submission and before we can proceed, we need you to address the following issues:

- 1. Thank you for stating the following financial disclosure:

  The study was conducted in the context of the CIRCLE trial. The Circle Study was funded by the NIHR HTA programme (ref 09/144/50). The views expressed are those of the authors and not necessarily those of the NHS, the NIHR or the Department of Health.

  Please state what role the funders took in the study. If the funders had no role, please state:

  "The funders had no role in study design, data collection and analysis, decision to publish, or preparation of the manuscript."

  If this statement is not correct you must amend it as needed.
  Please include this amended Role of Funder statement in your cover letter; we will change the online submission form on your behalf.

Response:

This has now been added to the cover letter. We have tracked the change.

- 2. Thank you for stating the following in your Competing Interests section:

  None

  Please complete your Competing Interests on the online submission form to state any Competing Interests. If you have no competing interests, please state

  "The authors have declared that no competing interests exist."

  as detailed online in our guide for authors at http://journals.plos.org/plosone/s/submit-now
  This information should be included in your cover letter; we will change the online submission form on your behalf.

Response:

This has now been added to the cover letter. We have tracked the change.

- 3. We note your current Data Availability statement is:

  - "No - some restrictions will apply"
  - "Data are interview transcripts that are not shared publicly. Authors will consider requests for access to the data."

  PLOS journals require authors to make all data necessary to replicate their study’s findings publicly available without restriction at the time of publication. When specific legal or ethical restrictions prohibit public sharing of a data set, authors must indicate how others may obtain access to the data. For more information, please see https://journals.plos.org/plosone/s/data-availability.

  Before we proceed with your manuscript, please address the following prompts:

  a.) If there are ethical or legal restrictions on sharing a de-identified data set, please explain them in detail (e.g., data contain potentially identifying or sensitive patient information, data are owned by a third-party organization, etc.) and who has imposed them (e.g., a Research Ethics Committee or Institutional Review Board, etc.). Please also provide non-author contact information* for a data access committee, ethics committee, or other institutional body to which data requests may be sent.

  *PLOS’ Data Policy states that authors cannot be the sole named individuals responsible for ensuring data access.

b.) If there are no restrictions, please upload the minimal anonymized data set necessary to replicate your study findings to a stable, public repository and provide us with the relevant URLs, DOIs, or accession numbers. For a list of recommended repositories, please see https://journals.plos.org/plosone/s/recommended-repositories. You also have the option of uploading the data as Supporting Information files, but we would recommend depositing data directly to a data repository if possible.

We will update your Data Availability statement on your behalf to reflect the information you provide.

Response

We have now added the following to the submission:

“Data are interview transcripts that are not shared publicly as they may contain potentially identifying information. Requests for access should be sent to circle.trial.ucl@gmail.com will be considered carefully.”

- a.) If there are ethical or legal restrictions on sharing a de-identified data set, please explain who has imposed them (e.g., a Research Ethics Committee or Institutional Review Board, etc.).

Response:

We received ethics approval for the present study was received as part of substantial amendment no. 6 to CIRCLE on 22nd November 2016 by the London – South East NRES committee (REC reference 11/LO/1939). Our ethics approval allowed for: "Interviews and focus groups will be digitally recorded and transcribed verbatim.” As such, while transcripts do not contain directly identifying information such as names, they have otherwise been transcribed verbatim. Given that it is a qualitative dataset involving one-to-one interviews and focus groups, it may be possible for a motivated individual to identify mental health teams and/or individual clinicians, particularly if they combined this dataset with other data/information sources. As such, we are mindful of our data handling and storage requirements under our ethics approval and under GDPR guidelines. Before releasing the dataset to someone, we and the recipient would need to ensure that all data handling and storage requirements have been met, and receive the required approvals for the transfer and work to be undertaken.

- b.) Please also clarify who is the recipient of the provided contact email "circle.trial.ucl@gmail.com" and what their relationship to the data is?
- c.) If the recipient is an author of this paper, please provide a non-author point of contact for a data access committee, ethics committee, or other institutional body to which data requests may be sent.

Response:

The Chief Investigator for the CIRCLE trial, in which context the present study was conducted, who is also the senior author on this paper is Prof. Sonia Johnson. She is the director of the Mental Health Policy Research Unit. The recipient of emails to "circle.trial.ucl@gmail.com" is the research administrator for the MHPRU. They are not connected to the present study and are not an author.

Thank you.

We've returned your manuscript to your account. Please resolve these issues and resubmit your manuscript within 21 days. If you need more time, please email the journal office at plosone@plos.org. We are happy to grant extensions of up to one month past this due date. If we do not hear from you within 21 days, we will withdraw your manuscript.

Please log on to PLOS Editorial Manager at https://www.editorialmanager.com/pone/ to access your manuscript. You will find your manuscript in the 'Submissions Sent Back to Author' link under the New Submissions menu. Be sure to remove your previous manuscript file if you are uploading a new file in response to these requests. After you've made the changes requested above, please be sure to view and approve the revised PDF after rebuilding the PDF to complete the resubmission process.

We are requesting these changes to comply with the PLOS ONE submission guidelines (https://journals.plos.org/plosone/s/submission-guidelines). Please note that we won't send your manuscript for review until you have resolved the above requests.

Thank you for submitting your work to PLOS ONE and supporting our mission of Open Science.


Kind regards,

Oriel Jerome Delas Alas Vida
PLOS ONE
